# Defining Early-Onset Colon and Rectal Cancers

**DOI:** 10.3389/fonc.2018.00504

**Published:** 2018-11-06

**Authors:** Daniel Jacobs, Rebecca Zhu, Jiajun Luo, Gabriella Grisotti, Danielle R. Heller, Vadim Kurbatov, Caroline H. Johnson, Yawei Zhang, Sajid A. Khan

**Affiliations:** ^1^Yale University School of Medicine, New Haven, CT, United States; ^2^Department of Surgery, Yale University School of Medicine, New Haven, CT, United States; ^3^Department of Environmental Health Sciences, Yale University School of Public Health, New Haven, CT, United States; ^4^Yale Cancer Center New Haven, CT, United States; ^5^Section of Surgical Oncology, Department of Surgery, Yale University School of Medicine, New Haven, CT, United States

**Keywords:** colorectal cancer, colon cancer, rectal cancer, early-onset colorectal cancer, SEER program, epidemiology, tumor pathology

## Abstract

**Background:** Colorectal cancer (CRC) incidence is rising in the young, yet the age of those affected is not clearly defined. In this study, we identify such cohorts and define clinicopathological features of early-onset colon and rectal cancers.

**Methods:** The Surveillance, Epidemiology and End Results Program (SEER) database was queried to compare clinicopathological characteristics of colon and rectal cancers diagnosed during 1973–1995 with those diagnosed during 1995–2014.

**Results:** We identified 430,886 patients with colon and rectal cancers. From 1973–1995 to 1995–2014, colon cancer incidence increased in patients aged 20–44 years, while rectal cancer incidence increased in patients aged ≤54 years. The percent change of cancer incidence was greatest for rectal cancer with a 41.5% (95% confidence interval (CI): 37.4–45.8%) increase compared to a 9.8% (CI: 6.2–13.6%) increase in colon cancer. Colon cancer has increased in tumors located in ascending, sigmoid, and rectosigmoid locations. Adenocarcinoma histology has increased in both colon and rectal cancers (*P* < 0.01), but mucinous and signet ring cell subtypes have not increased (*P* = 0.13 and 0.08, respectively). Incidence increases were race-specific, with rectal cancer seeing similar rises in white (38.4%, CI: 33.8–43.1%) and black populations (38.0%, CI: 26.2–51.2%), while colon cancer as a whole saw a rise in white (11.5%, CI: 7.2–15.9%) but not black populations (−6.8%, CI: −14.6–1.9%).

**Conclusions:** Our study underscores the existence of key differences between early-onset colon (20–44 years) and rectal cancers (≤54 years) and provides evidence-based inclusion criteria for future investigations. We recommend that future research of CRC in the young should avoid investigating these cases as a single entity.

## Background

Colorectal cancer (CRC) is the third most commonly diagnosed cancer in the United States, and is the third leading cause of death in both men and women (1). In 2018, there will be an estimated 140,250 new cases and 50,630 deaths attributed to the disease (1). While CRC remains highly prevalent in the United States, most age groups over 50 years have seen substantial declines in its incidence since its peak in 1985 when ~138,000 new cases and 60,000 deaths were attributed to the disease (2, 3). This fall in incidence preceded current population screening guidelines recommended by the United States Preventative Services Take Force (USPSTF) in 1995 and has been explained by increased early detection and removal of pre-malignant polyps, decreased usage of tobacco products, and to a lesser extent improvement in diet, physical activity and weight control (4–8).

Despite a general decline in the number of new cases, CRC has been increasing in incidence in young adults (3, 9). This trend was clearly established by the early 2000's, and since then, an extensive amount of research has been performed to understand the affected patient demographics and tumor characteristics (10). Importantly, most patients with early-onset CRC have sporadic disease, with 20–30% having a hereditary genetic predisposition, such as a familial adenomatous polyposis, Lynch syndrome or a positive family history (11–13).

To address why the incidence of sporadic early-onset CRC is increasing, many groups have explored the demographic and lifestyle factors that place young patients most at risk (11, 14). These include being of black race, of lower socioeconomic background, or having modifiable risk factors, such as smoking, low fiber diet, or sedentary lifestyle (7, 14). However, recent evidence raises questions about whether these modifiable factors are causative (15). Other studies have examined differences in clinical presentation and tumor characteristics between younger and older patients (14, 16). Genomic profiling has shown that younger patients with CRC are more likely to have microsatellite instability or BRAF mutations associated with microsatellite stability (17). Work to further identify genetic mutations and RNA expression changes in early-onset CRC tumors is an active area of research (18, 19).

While investigators elucidated differences contributing to the rising incidence of early-onset CRC, few studies have tried to systematically define “early-onset.” Many groups have designated these individuals to be under the age of 30, 40, or 50, while others have used the NCI definition of adolescents and young adults (AYA) (14, 17, 20–22). Despite mounting evidence that colon and rectal cancers have increased at variable rates in different age groups, few groups justify their choice of reference age range and most use a single cut off age (3). With different research groups using variable age groups or combining colon and rectal cancers in the study design, results among studies are difficult to interpret and compare.

In this study, we sought to define “early-onset” based on incidence trend data and to propose unique age ranges for early-onset colon cancer and early-onset rectal cancer. We further wanted to see whether the clinicopathological features of early-onset colon and rectal cancers shared common trends to better appreciate if the rise in tumor burden stems from a common pathogenesis. Finally, we asked whether racial disparities exist between early-onset colon and rectal cancers. The objective of addressing these questions was to aide in guiding future research methodology and to understand how to more effectively screen for CRC in patients currently below the current recommended screening ages.

## Methods

### Incidence of colon, rectal, and colorectal cancer

The Surveillance, Epidemiology and End Results Program (SEER) database consisting of 9 registries (Atlanta, Connecticut, Detroit, Hawaii, Iowa, New Mexico, San Francisco-Oakland, Seattle-Puget Sound, and Utah) from 1973 to 2014 was queried for incidence rates of colorectal, colon and rectal cancers. Rates were standardized to the 2010 US standard population. Rate ratios comparing incidence in 1995–2014 to incidence in 1973–1994 were calculated using SEER^*^Stat software Version 8.3.4 (https://seer.cancer.gov/seerstat/), and 95% confidence intervals (CIs) were calculated using the Tiwari modification. Ratios were converted to percentages. The year 1995 was chosen because this is the year in which the USPSTF introduced formal screening guidelines for colorectal cancer screening (8). Patients who developed colorectal cancer sporadically or as part of a hereditary or known genetic condition were all included in the analysis. Only tumors with malignant behavior were selected. Tumors of the appendix were excluded. Colorectal cancer is defined as the cancer of the colon, rectosigmoid junction, and rectum. Colon cancer includes the colon and rectosigmoid junction. Right-sided colon includes the cecum, ascending colon, hepatic flexure and transverse colon. Left-sided colon includes the splenic flexure, descending colon, sigmoid colon and rectosigmoid junction. Ages represent “Age at Diagnosis.”

The SEER database is a publicly available large, population-based cancer registry database managed by the National Cancer Institute. The 9 registries that were queried for this analysis covers 9.4% of the United States population based on the 2010 census data, and is largely representative of the United States demographic composition (https://seer.cancer.gov/registries/data.html). Information collected from the database includes patient demographics, tumor characteristics, cancer staging, treatment, and outcomes (23). The SEER database is used by many research groups to trend incidence and mortality rates over time, and is the database utilized for yearly reports on cancer statistics (1).

### Analyzing tumor characteristics

Data was obtained and analyzed from the SEER database using SEER^*^Stat as previously described. Colon and rectal cancer pathologic staging from 1988 to 2014 was obtained by merging the American Joint Committee on Cancer (AJCC) 3rd and 6th editions. No significant difference in staging of colon and rectal cancers precluded such merging. “Stage 0–2” is defined as any T, N0, M0 tumors; “Stage 3” is defined as any T, N1, M0 tumors, and “Stage 4” is defined as any T, any N, and M1 tumors. Tumor histologic types were obtained from the Classification for Disease for Oncology 3rd edition (ICD-O-3) in the SEER^*^Stat program, and the codes queried for both colon and rectal cancers were similar to those used in other published research (24). Histologic types included adenocarcinoma (codes 8050–8052, 8140–8148, 8210–8231, 8255–8263, 8510, 8560–8576), mucinous adenocarcinoma (codes 8480–8481), and signet ring cell carcinoma (code 8490). Tumor grade was defined by AJCC guidelines.

### Analyzing demographics

All races were included in the data analysis. However, when patient race was specifically analyzed, only “White” and “Black” populations were selected for from the “Race Recode (White, Black, Other)” variable as defined by SEER^*^Stat.

### Ethics statement

This study was reviewed by and approved by the IRB at Yale University. Data was obtained from an anonymous publicly available database, so no consent was needed.

## Results

**Figure 1:**
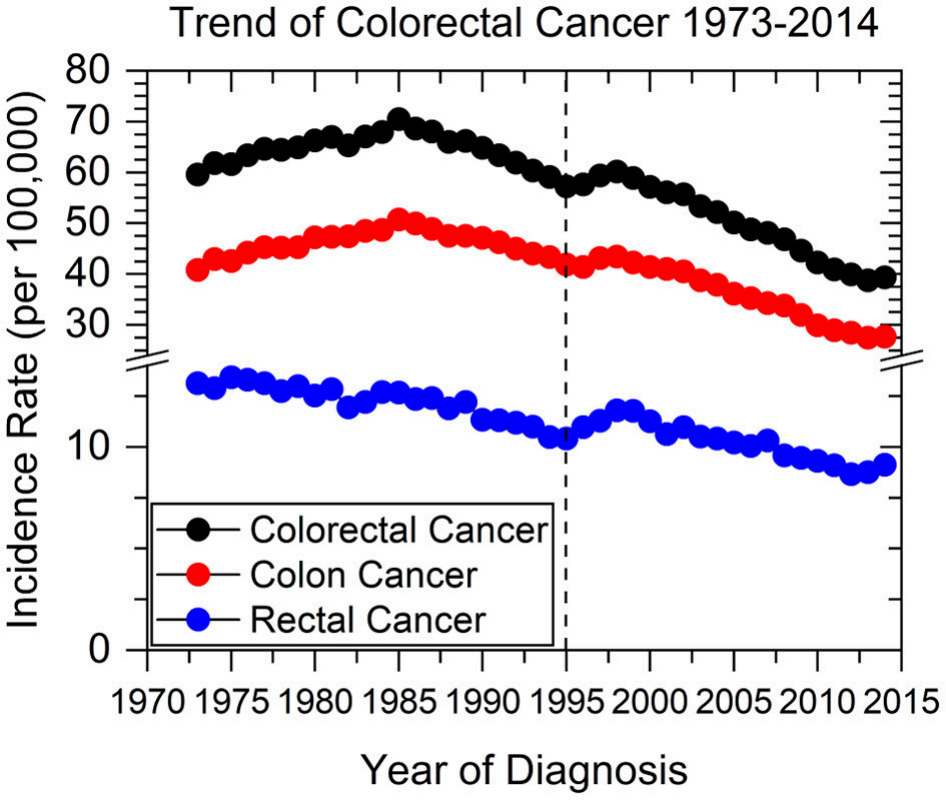
Colorectal, colon, and rectal cancer incidence rates from 1973 to 2014. Dashed line represents when current screening guidelines were recommended in 1995 by the USPSTF.

We identified 430,886 patients with colorectal cancers using the SEER database from 1973 to 1995. Collectively, the overall incidence of CRC has been declining since 1985, when incidence rate peaked at >70 new cases per 100,000 persons (Figure 1). Colon cancer specifically has followed this trend, while rectal cancer incidence has been decreasing since SEER data collection began in 1973. In 1995 there was a sharp incline in the incidence of both colon and rectal cancers, peaking between 1997 and 1998.

**Figure 2:**
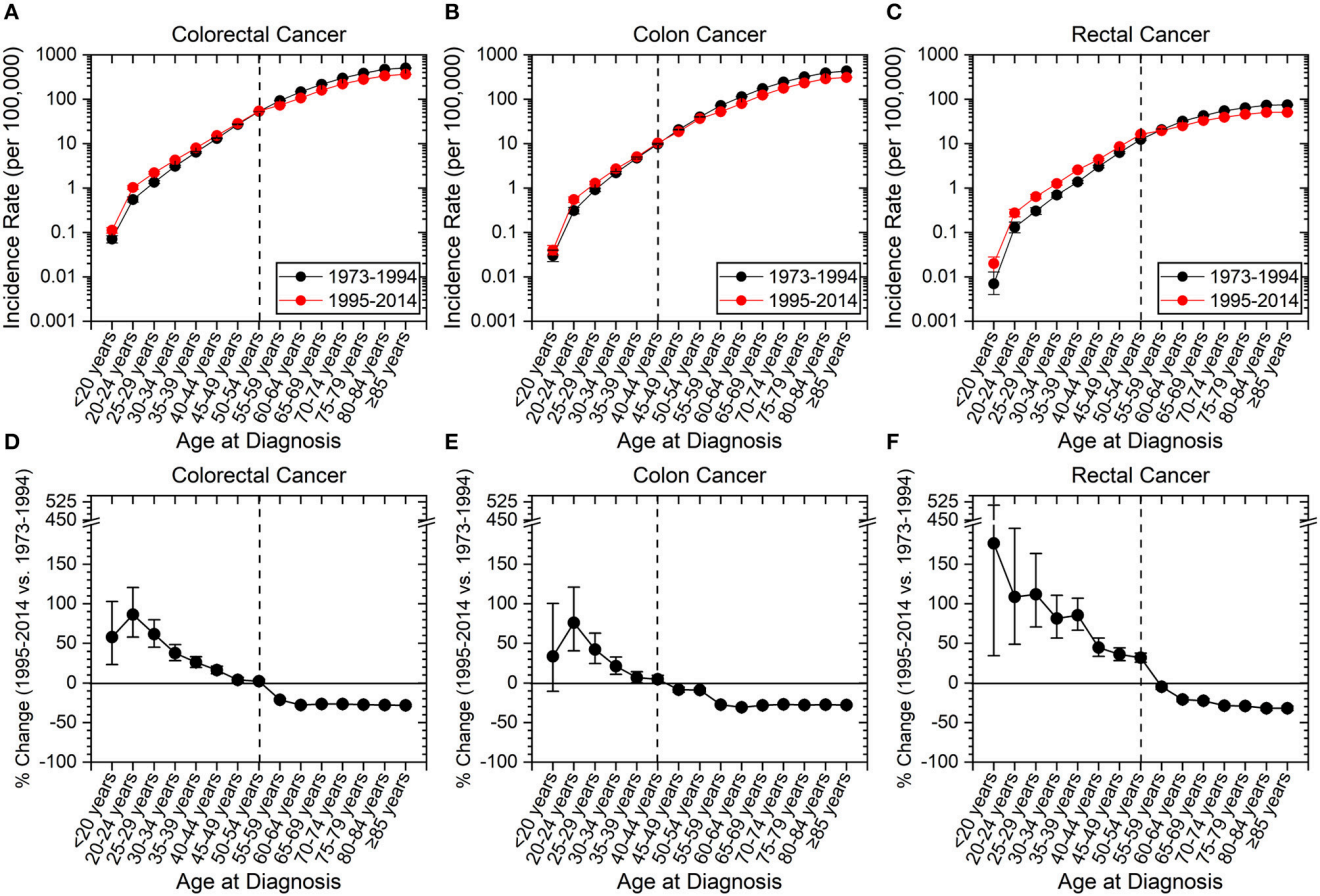
Incidence rates of colorectal, colon and rectal cancers expressed as absolute numbers and as a percentage change from 1973–1994 to 1995–2014. Error bars represent 95% confidence intervals. Dashed line is to guide the eye to age range at which change in incidence rate converts from positive to negative. **(A–C)** Incidence rate of cancer as a function of site. **(D–F)** Percentage change of cancer incidence as a function of site. **(A–F)** Younger patients have seen an increase in cancer incidence, while older patients have seen a decline. Rectal cancer has seen the highest amplitude change compared to colon cancer and has a later age for transition from a positive to negative percentage change.

The incidence of CRC has increased in those aged <55 years and decreased in older individuals (age ≥55 years) between the time periods of 1973–1994 to 1995–2014 (Figures 2A,D). Colon cancer has increased in incidence in patients aged 20 to 44 years (Figures 2B,E), and rectal cancer has increased in incidence in those <55 years (Figures 2C,F). Hereon, “early-onset colon cancer” and “early-onset rectal cancer” are defined by these aforementioned age ranges, respectively. Strikingly, there was >100% increased incidence for rectal cancers for patients aged <20 years (176.5%, CI: 34.4–511.3%), 20–24 years (108.6%, CI: 48.7–195.4%), and 25–29 years (111.8%, CI: 70.8–163.7%). The overall increase in early-onset rectal cancer was 41.5% (CI: 37.4–45.8%), while the overall increase in early-onset colon cancer was 9.8% (CI: 6.2–13.6%). In contradistinction, there has been a near uniform decrease in colon and rectal cancer incidence of >20% for those aged 60 and older since 1995.

**Figure 3:**
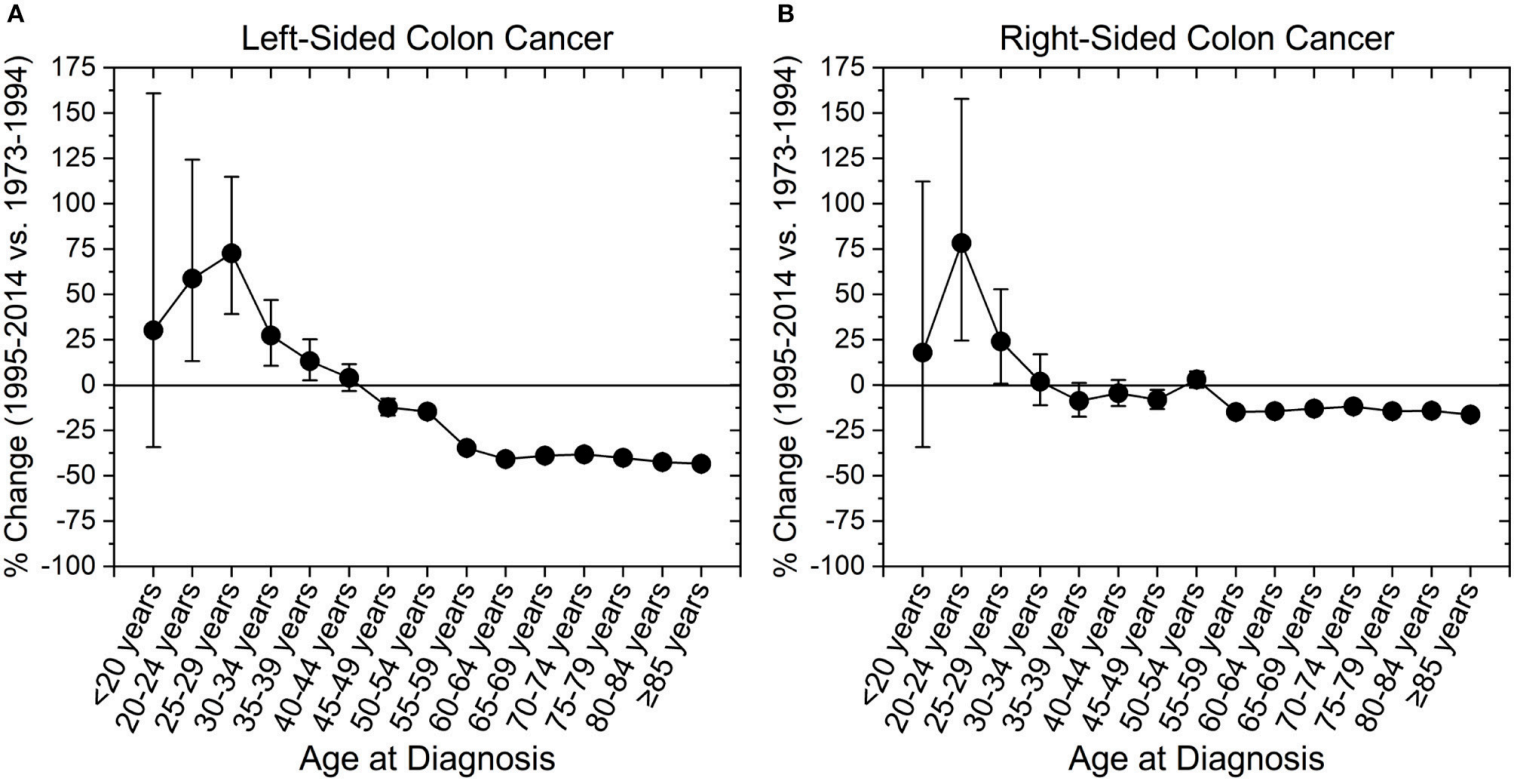
Percent change in colon cancer incidence rate by colon side. Error bars represent 95% confidence intervals. **(A)** Left-sided colon cancer increased in incidence among those aged 20–39 years, while **(B)** right-sided colon cancer increased in those aged 20–29 years.

**Figure 4:**
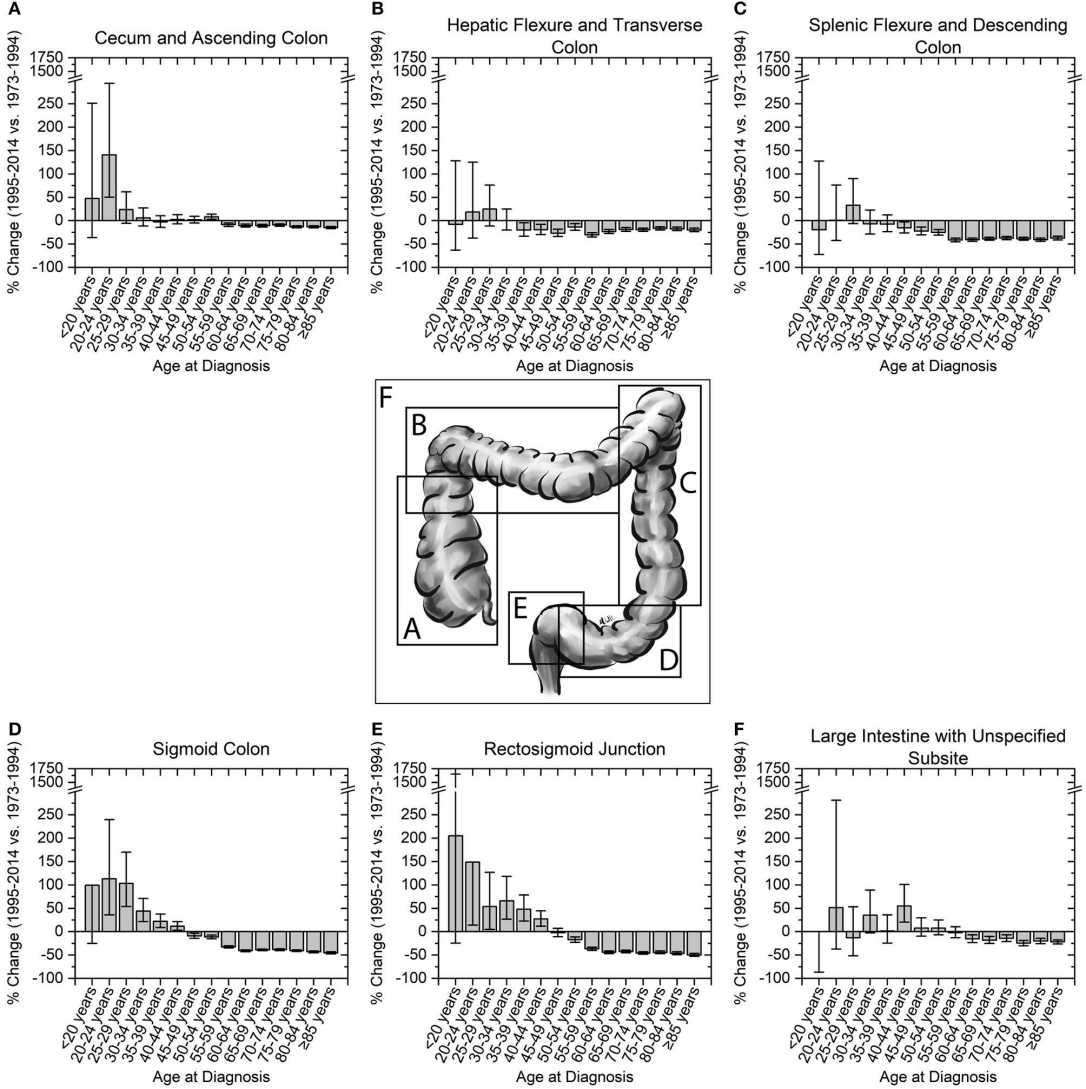
Percent change in colon cancer incidence rate by colon subsite. Error bars represent 95% confidence intervals. **(A,B)** Right sided colon cancers, and **(C–E)** left-sided colon cancers are broken into their respective colon subsites. **(F)** Cancers of the large colon with unspecified subsite show a significant increase in incidence in patients aged 40–44 years.

We next assessed incidence trends in anatomical location of colon cancer. Interestingly, trends were different for right- and left-sided tumors (Figure 3). Right-sided tumors have significantly increased in patients 20–29 years old, while for left-sided tumors the increase extended to patients 39 years old (Figure 3). To further understand the rise in right- and left- sided colon cancers, we next analyzed the change in incidence for individual colon segments (Figure 4). The increase in incidence in right-sided colon cancers was largely driven by tumors arising in the cecum and ascending colon, with percent increases as high as 140.1% (CI: 50.4–294.3%) for patients aged 20–24 years. Both cecal and ascending colon cancers had statistically significant increases in incidence in this age group independently (data not shown). The incidence in sigmoid cancer increased by roughly 100% for patients ≤29 years and by >100% for patients ≤24 years with rectosigmoid cancers. No significant increase in cancer incidence was noted in the hepatic and splenic flexures, transverse colon, or descending colon. Cancers of the large intestine with unspecified subsite had a statistically significant increase in incidence in patients aged 40–44 years (Figure 4F). This may explain why although colon cancer overall has increased in ages 20–44, neither right nor left-sided colon cancers saw a rise the 40–44 year age group.

**Figure 5:**
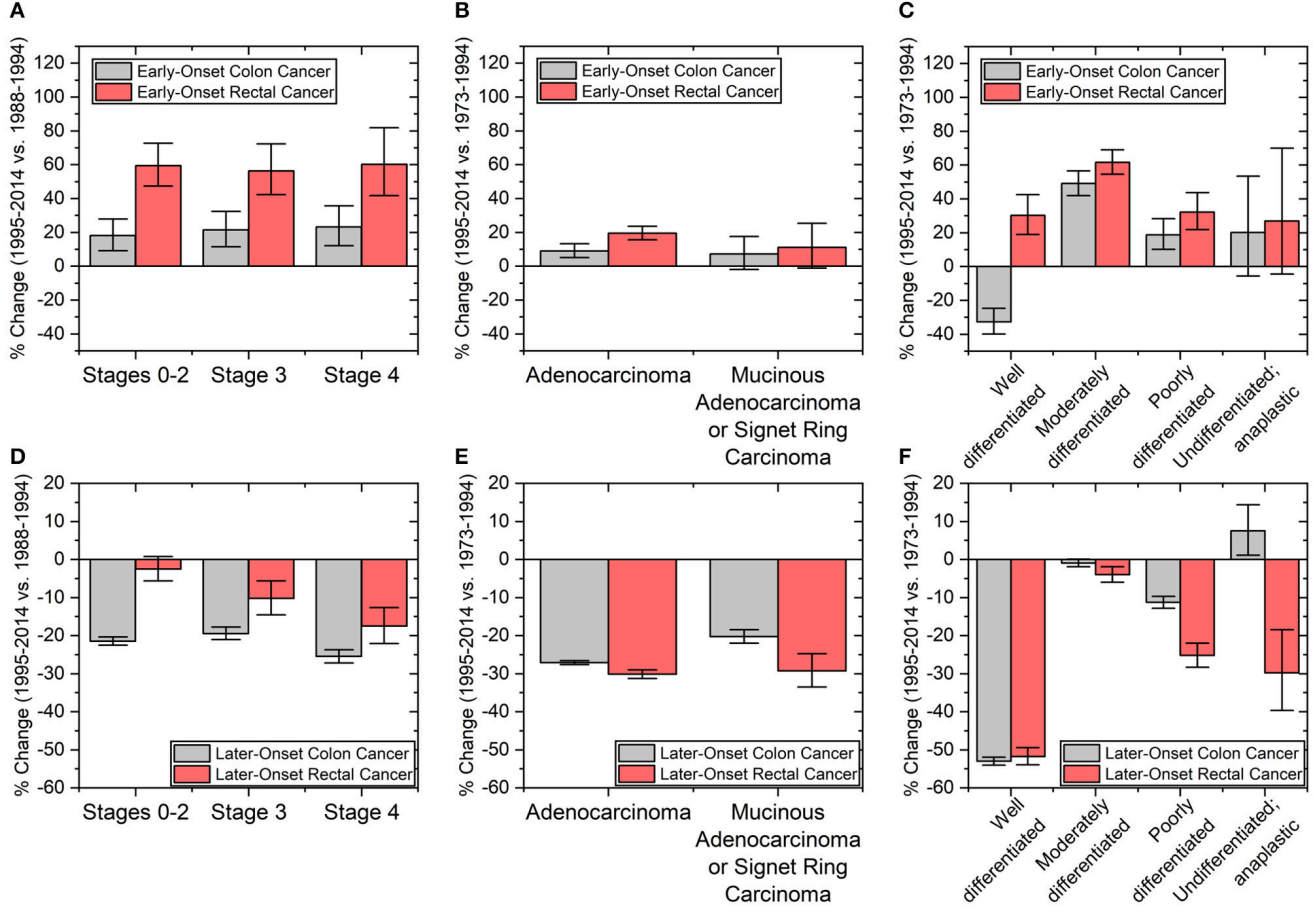
Percent changes in patients with **(A–C)** early onset colon (ages 20–44 years) and rectal cancers (ages ≤54 years) and **(D–F)** later-onset colon and rectal cancers (defined as age ≥60 years old). Clinicopathological tumors characteristics include **(A,D)** cancer staging and tumor **(B,E)** histology and **(C,F)** grade. Staging based on a merging of AJCC 3rd and 6th editions from the time periods of 1988–1994 to 1995–2014. Error bars represent 95% confidence intervals.

The trend of colon and rectal cancers appears to depend on the site of the primary tumor. We therefore investigated whether tumor characteristics were similar or different between early-onset colon and early-onset rectal cancers. Cancers that were node-negative (Stages 0–2), node positive (Stage 3), or metastatic (Stage 4) increased in incidence by the same degree within early-onset colon and early-onset rectal cancers, respectively (Figure 5A). However, the overall percentage rise, as noted before, was larger in early-onset rectal cancer than early-onset colon cancer (58.7 vs. 20.9%, *p*-value < 0.01 using 1-tailed heteroscedastic student *t*-test). With regard to microscopic examination of tumor histologic type, both early-onset colon and rectal cancers saw increases in adenocarcinoma (*p*-values both < 0.01; Figure 5B), but no statistically significant increase in the incidence of mucinous adenocarcinoma or signet ring cell carcinoma (*p*-values 0.13 and 0.08, respectively). Tumors in early-onset colon and rectal cancer were also similar they both had the largest incidence change of moderately differentiated tumors (49.1%, CI: 42.0–56.6% and 61.6%, CI: 54.5–69.1%, respectively; Figure 5C). Well-differentiated tumors had decreased incidence for early-onset colon cancer but increased incidence for early-onset rectal cancer. Neither early-onset colon nor rectal cancer saw statistically significant changes in undifferentiated tumors (*p*-values 0.14 and 0.10, respectively). These increases in tumor biological features over time stand in stark contrast to the major declines in the same features in patients with colon and rectal cancers aged >60 years (Figures 5D–F). Finally, we wanted to see how sex, stage, grade and histology varied by colon side. Interestingly, there was no significant difference in the incidence rate change in these characteristics when directly comparing early-onset right-colon and left-colon cancers (Supplementary Figure 1).

To identify which patient demographics have seen the greatest rise in colon and rectal cancers, we explored how trends varied by race. Early-onset colon cancer has risen in incidence more in white populations (11.5%, CI: 7.2–15.9%) than in black populations (6.8%, CI: −14.6–1.9%). However, for early-onset rectal cancer both white and black populations have seen similar changes (38.4%, CI: 33.8–43.1% vs. 38.0%, CI: 26.2–51.2%). To further support the observation that the increase in early-onset colon cancer may be more due to rises in incidence in white populations compared to black populations, the ratio of number of cases for either race was computed. From 1973 to 1994, the rate for developing early-onset colon cancer was 53.4% (CI: 42.3–65.1%) higher in black populations than white populations. However, in 1995–2014, this value had dropped to 28.3% (CI: 20.6–36.3%). Surprisingly, the data also showed that white populations had seen greater decreases in colon and rectal cancers compared to black populations in those over the age of 60.

## Discussion

While the overall incidence of CRC has been decreasing since 1985, there has been a paradoxical rise in young adults. In this study, we identified never reported clinicopathologic details associated with this change. Colon cancers have increased in incidence for individuals aged 20–44 years, while rectal cancers have increased for individuals ≤54 years. We also showed that the rise in colon cancer was mostly attributed to tumors of the ascending colon, sigmoid colon, and rectosigmoid junction. Further, we demonstrated that changes in incidence rates of early-onset colon and rectal cancers affected white populations more than black populations.

A key question underlying early-onset CRC research has been whether tumors currently diagnosed share a common pathogenesis to CRCs from an earlier era or whether there exists a new and distinct process. This question was recently raised by Yeo et al. and underpins contemporary genetic studies and relies on the ability to separate these “new” cancers from the distribution of “classic” cancers have continued to exist (15, 18). The best way to approach this problem is through evidence-based methodology that samples patients from age groups that are more likely to have a “new” tumor variant over a “classical” one. Therefore, while researchers have traditionally used the cut-off of patients ≤40 or ≤50 years to study early-onset CRC as a single disease entity, such cut-offs are blunt tools that likely reduce the specificity of findings obtained (10, 13). Our results may be used as a tool to identify these patients. Moreover, we underscore that colon and rectal cancers are have distinct age range profiles.

Our analysis centered around identifying changes in incidence rates of colon and rectal cancers for different age groups since the introduction of formal screening guidelines by the USPSTF in 1995. We calculated incidence rates using the SEER database from before the introduction of formal CRC screening guidelines (1973–1994) to the years since (1995–2014). This analysis allowed us to see the effectiveness of such recommendations in every age group. The methodology differs from the traditional approach of using the annual percentage change (APC) to calculate changes in incidence (3, 25). Nonetheless, we believe that comparing two time periods to be a more robust measurement as it increases the effective sample size and smoothens small temporal fluctuations.

While small fluctuations are averaged out in our method, we recognize that caution in interpretation is needed when fluctuations are large, such as may be the case for colon cancer in patients aged 40–49 and 50–54 years. According to Siegel et al. these age groups saw a statistically significant increase in APC in 1994 and 1996, respectively (3). We do not dispute this finding, which may be particularly useful in determining when such groups cross the “incidence rate threshold” that qualifies them for inclusion in screening guidelines. Compared with higher resolution APC analyses, we believe our analysis to be a holistic interpretation of the demographic, clinical, and pathologic landscape of CRC diagnosis. We emphasize that our data is otherwise congruent with other published research, which we believe adds validity and generalizability to our findings.

Multiple recent studies have shown that earlier-onset CRC tends to disproportionately arise from the left colon and rectum (13–16). These studies compare disease prevalence in earlier-onset individuals and later-onset individuals to estimate cancer burden. Our data support that cancer at these sites is not only more prevalent but has also been increasing in incidence over a broad range of ages (3, 26). While its prevalence may be higher, left-sided cancer may be increasing in incidence at a slower rate than right-sided cancer, especially cancers of the cecum and ascending colon for patients aged 20–24 years old (140.1% increase, CI 50.4–294.3%). A rise specifically in cecal and ascending colon cancer incidence has not been previously reported and is of particular clinical importance, since right-sided cancers have unique histopathological characteristics and tend to carry a worse prognosis (27–30).

Our work does not directly address how modifiable or environmental risk factors may influence the development of early-onset colon and rectal cancers. Despite the fact that early-onset colon and rectal cancers have a higher absolute incidence in black populations, our study notes a previously-unrecognized trend that colon cancer is rising fastest in white populations (31). This trend makes it important to consider potential causes for this disproportionate effect. One theory might include associations with inflammatory bowel diseases including ulcerative colitis or Crohn's disease, both of which have increased more in white populations and are risk factors for developing CRC (32, 33). We also propose the theory that white populations may have better access to health care than black populations, and therefore have a higher rate of diagnosis. This theory may supported by research that shows that the uninsured, who are more likely to be black than white, tend to present at later cancer stages (34). Furthermore, extrapolating from adult data, patients who are white, have higher incomes, born in the United States, are well-educated, and have private insurance or Medicare are more likely to be undergo recommendation-consistent screening than others (35, 36). Moreover, in 1997 there was no difference in screening adherence between white and black populations, but recent data has shown disparities (35, 37). Finally, apart from fiber and fat content, diets that alter the community structure of one's microbiome including a shift toward overabundance of *Fusobacterium nucleatum*, or degree of inflammation, may change the pathogenesis of disease (38–40).

Translation of epidemiological trends to real-world application remains a goal of our study. Recent modeling work has predicted that screening the general public under the age of 50 may cause more harm than good (41, 42). These studies are of great importance as the American College of Gastroenterology has recently recommended screening African Americans starting at age 45, and the American Cancer Society recommending general population screening starting at age 45 (43, 44). Such debates in the scientific field seem likely to continue, so further data is needed to make evidence-based decisions and recommendations. Based on our results, we recommend models for screening should be updated over time to take demographics, including race, into account and should also use data on colon site-specific incidence rate changes to determine the utility of different endoscopic screening technologies.

Differences in colorectal screening practices and adherence for different populations was considered a potential explanation for differences in cancer incidence based on tumor site, stage, and population. Our data suggests that screening for early-onset CRC does not explain the rise in the cancer incidence in these groups since stage 0–2 cancers increased at the same rate as stage 3 and stage 4 cancers. If screening were to be the reason, we would have expected to see a disproportionate rise in early-stage cancers. Furthermore, screening has not been recommended in patients in the age groups we propose for early-onset colon and rectal cancers, except in patients with hereditary cancer syndromes or a first degree relative. It is possible that the greater rise in rectal cancers compared to colon cancers may be due to use of sigmoidoscopy as compared to colonoscopy. However, there is no evidence to support this, as sigmoidoscopy has been decreasing in use over the past few decades from 9.4% in 2000 to <1% in 2015 (35, 36). Finally, the increased rate of early-onset colon cancer in white populations may be explained by better access to care, as discussed above. On the other hand, with regards to patients with later-onset disease, decreased incidence of stage 3 and 4 colon and rectal cancers is consistent with the general idea that screening reduces the incidence of colorectal cancers in older patients (Figure 5).

Our work has several limitations. One of the main limitations of the study is that the AJCC pathologic staging for colorectal cancers in the SEER database started from 1988. Since all of our other analysis started in 1973, 15 years-worth of patient information was not included in the baseline measurement from which to compare the rate of developing differently staged tumors during the time period of 1995–2014. Second, since the SEER database used in this analysis captures ~10% of the general population, restricted by the states in which registries exist, sampling bias may exist in that the population being studied does not exactly represent the United States as a whole. This prominently includes potential overrepresentation of foreign born patients and those who live in urban areas (23). Finally, we could not analyze whether Hispanic ethnicity influenced trends of colon and rectal cancers as this was not a population variable in the SEER database and therefore could not be used to calculate incidence rates.

In conclusion, we show that early-onset colon and rectal cancers encompass different patient age ranges and outcomes vary by tumor location and race, which suggests that differences in pathogenesis exist between the two diseases despite potentially similar histopathological characteristics. Our work has important implications, including proposing a standard set of age definitions for early-onset colon cancer (20–44 years) and early-onset rectal cancer (≤54 years), respectively, and identifying at-risk colon segments in younger populations that may further inform screening practices. A consistent set of definitions among researchers will provide clarity to future studies in early-onset cancers.

## Author contributions

DJ, RZ, GG, DH, VK, CJ, YZ, and SK: conceptualization; DJ, RZ, JL, GG, DH, VK, YZ, and SK: methodology; DJ, RZ, JL, YZ, and SK: software; DJ, JL, YZ, and SK: validation; DJ, YZ, and SK: formal analysis; DJ and SK: investigation; DJ, RZ, JL, GG, YZ, and SK: resources; DJ: data curation; DJ, YZ, and SK: writing–original draft; DJ, RZ, JL, GG, DH, VK, CJ, YZ, and SK: writing–review and editing; DJ, GG, DH, VK, YZ, and SK: visualization; DJ, CJ, YZ, SK, YZ, and SK: project administration; DJ and SK: funding acquisition.

### Conflict of interest statement

The authors declare that the research was conducted in the absence of any commercial or financial relationships that could be construed as a potential conflict of interest.
