# Supplementary material for: Defining Early-Onset Colon and Rectal Cancers
Source: Front Oncol. 2018 Nov 6;8:504. doi: 10.3389/fonc.2018.00504 (PMC6232522; doi:10.3389/fonc.2018.00504)
Supplement: Supplementary file 1 [file Data_Sheet_1.docx]

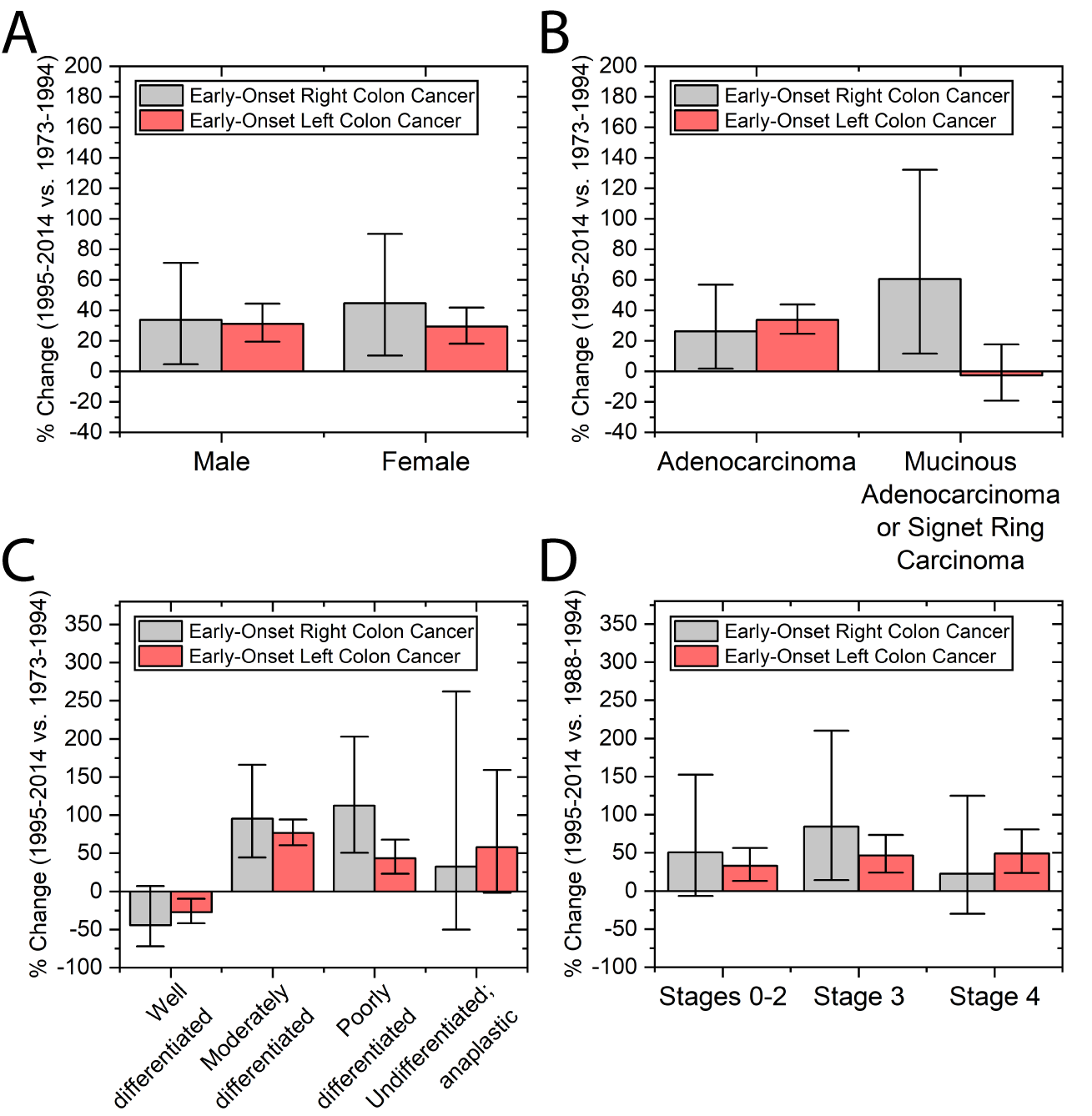


Supplemental Figure 1. Comparison of the percent change in incidence for early-onset right-sided versus left-sided colon cancers, which were defined as by age ranges 20-29 and 20-39, respectively. Direct comparison of early-onset right- versus left-sided colon cancer did not show a difference in distribution with regard to *A*) sex, *B*) tumor grade, *C*) tumor histologic type, or *D*) cancer stage. Error bars represent 95% confidence intervals.
